# Where is the Child in Family Therapy Service After Family Violence? A Study from the Norwegian Family Protection Service

**DOI:** 10.1007/s10591-014-9323-5

**Published:** 2015-01-21

**Authors:** Anna Margrete Flåm, Bjørn Helge Handegård

**Affiliations:** 1 Institute of Psychology, UiT The Arctic University of Norway, Huginbakken 32, 9037 Tromsö, Norway; 2 RKBU – North, UiT The Arctic University of Norway, Tromsö, Norway

**Keywords:** Child therapy, Family therapy, Domestic violence, Family violence, Child maltreatment, UN Convention on the Rights of the Child

## Abstract

Extensive documentation on consequences of family violence laid the ground for a politically decided mandate for the Norwegian Family Protection Service (FPS) to prioritize families with children and violence. This study explores the practice of one of the country’s larger FPS offices following this mandate and its kick-off start. Data from all cases in 1 year with families with children and violence were gathered (106) as to what were cases referred, services provided, main cross-points, dilemmas, and challenges. Descriptive statistical analyses were utilized and qualitative analysis conducted. The study shows success in supplying a direct, much used route both for private persons and main collaborative agencies, although all abusers need others as promoters for change. The service succeeds to pioneer brief treatment combined with taking a stand against violence. However, while services are provided fairly quickly when violence is reported, several changes are called for: A more violence-sensitive intake procedure, stronger cooperation with specialty mental health service and primary health service, extended use of assessment tools and outcome measures. Given the nature of violence, particularly follow up measures are required. However, first and foremost, the study calls for a better inclusion of the child. Despite mandated priority, a major neglect of children takes place. In line with the UN Convention on the Rights of the Child, the Norwegian Family Protection Services in a country complying with this Convention is obliged to take the child more successfully into account in its own right. Future efforts are required to safeguard child-focused services.

## Introduction

Providing access to psychological treatment services for children and their caregivers after domestic violence is a general challenge. Even in a Norwegian context, with one of the strongest public health and welfare systems in the world, a critical view is needed of how professional services meet the treatment needs of those involved. Established professional habits may hinder seeing what are benefits or perhaps main gaps to be aware of in living, ongoing practice. In this study we explore the public Norwegian Family Protection Service (FPS) which has a mandated priority to provide specialized psychological treatment in cases where children live with violence in the family. We explore the structure, benefits, and challenges of this service, and discuss what implications can be drawn to strengthen such services.

Many studies show the frequency of domestic violence (Gilbert et al. 2009a; Thoresen and Hjemdal 2014), the consequences on the health and developmental well-being of children and young people, as well as on the capacity of adults for taking sufficient care (ACE-study 2013; Anda et al. 2006; Evans et al. 2008; Geffner et al. 2003; Gilbert et al. 2009a, b; Lanius et al. 2012a, b). The need for access to psychological treatment for the involved family members is well documented (e.g. Holt et al. 2008; Patterson and Vakili 2014; Read and Bentall 2012; Siegel 2013).

However, clinical research and literature point to many family therapy providers having been too hesitant to include children as part of the relevant collaborations, stating that despite the advocacy to include children, the youngest members of the family have often been excluded and more likely a topic talked about than active participants (Hartzell et al. 2009; Rober 2008; Ruble 1999; Sori and Sprenkle 2004). This tendency has been noticeable when issues relate to violence (Heltne and Steinsvåg 2010, 2011; Siegel 2013). In general, clinical research and literature point to a dichotomy between, on the one side, the family therapy traditions accused of ignoring the child and oversimplifying its intrapsychic processes, and, on the other side, the child psychiatry approaches accused of seeing the child isolated from its environment, thus individualizing and pathologizing the child’s problems (Lund et al. 2002).

As a parallel, substantial studies in Norway document the overall difficulties and almost blindness in the specialty mental health service for children and adolescents in perceiving children and youth’s experiences of violence, thus excluding such experiences from informing important treatment processes (Ormhaug et al. 2012; Reigstad et al. 2006; Røberg 2011; Wassnes 2012). Reluctance in the same services to include children’s families as part of ongoing work (Reigstad 2012), highlights the consequent risks of neglecting children’s experiences of violence, underestimating their need for family support, and not creating sufficient space for families to participate. Thus, a double risk turns up: fragmenting the child’s experiences and minimizing the family’s importance.

In a Norwegian context, such documented shortcomings stand out as a paradox. That is, major political plans and strategies are elaborated across governmental departments with the exact aim to provide treatment services *both* to children exposed to violence *and* to their caregivers (Ministry of Children, Equality and Social Inclusion 2013), *and* to collaborate across agencies (Ministry of Health Care Services 2009). But paradoxically, children still “fell through the cracks”. To tighten such pitfalls, additional measures have been taken: Children Advocacy Centers and National and Regional Competency Centers for Violence and Traumatic Stress were established and the Child Protection System (CPS) and Family Protection Services (FPS) strengthened.

## The Family Protection Service (FPS) as a Main Part of the Public Service Web at Family Violence

The Norwegian FPS’s general mandate is to provide specialized treatment for relational problems and crises. As a public service, its obligation is to supply treatment to single persons, couples, families, and children. This is either obtained by people referring themselves, by other services’ recommendation, or by the CPS mandating specialized treatment from the FPS in order to safeguard and secure necessary child protection. Formalized by law in 1998, the FPS is free of charge, with open access and no precondition of being referred by other agencies—that is, a so-called “low threshold” service. The only exception is the CPS’s judicial possibility to mandate treatment. Financed by the state, the service is organized with in total 52 offices (Jensen 2013), with approximately 3 offices in each of the 19 counties in the country to provide services where people live. The professionals are mainly psychologists, social workers, specialized teachers, some psychiatrists; most of them working under an umbrella of different systemic therapy approaches. Most professionals are certified family therapist according to credential programs of Norwegian schools and educational organizations (Jensen 2013). Initially, when the FPSs were established, most clients came for partnership difficulties. From 2007, a compulsory Mediation Institute was added, defined as a compulsory negotiation ritual for all parents in Norway to mediate arrangement for their children’s care and custody after parental divorce. Recently, the state authorities mandated a new obligation, to prioritize risk cases, defined as cases in which there are concerns about child neglect and violence against children (Norwegian Directorate for Children, Youth and Family Affairs 2014).

This new priority represents a major thematic shift consistent with a growing awareness, politically and professionally, of the prevalence of domestic violence and its major consequences, especially for involved children. The new mandate relates to major changes in the general society in Norway: a political awareness in the 70s of domestic violence, the subsequent development of the crisis centers throughout the country, the development of treatment services for men committing violence, and the following growing awareness of child sexual abuse, violence, and maltreatment. These changes laid the ground for a subsequent incorporation of the UN Convention on the Rights of the Child into Norwegian law (1999) to guarantee children rights to live without violence and for the views of the children to be given due weight in all matters affecting them.

## A Kick-Off Project in the FPSs

In the FPSs, this thematic shift got a kick-off by a goal-directed project implemented from 2004 until 2007, and thereafter prolonged through 2010. Initiated by Minister Laila Dåvøy at the Ministry of Children, Equality, and Social Inclusion and funded by the national government at the time, the project “Children Living in Families with Violence” was mentored by two professional institutions, The Alternative to Violence (ATV), Oslo, and the Centre for Crisis Psychology (SfK), Bergen. Nine FPS offices throughout the whole country took part. The aim was to strengthen the knowledge, the organization, and the methodological capacity of the FPSs to offer treatment to families with children and violence. Knowledge development was secured through seminars and clinical supervision arranged for partaking offices continuously throughout the project period (Heltne and Steinsvåg 2010), however, without including any cut-off for pre- or post-criteria of the services.

Although definitions of domestic violence vary (Gilbert et al. 2009a; Krug et al. 2002), the one used in the kick-off project was: “Any actions directed towards another, that, by harming, injuring, frightening, or insulting, makes this person do something against his/her will, or abandon doing something that he/she wants” (Isdal 2013). It is, however, clear that where children are involved, the definition has to be expanded (MacMillian et al. 2013): violence in the family strikes the home as the most important developmental arena for attachment and trust. Children are forced to live with a lack of security, support and comfort from their main caregivers. The same persons engaged in violence abandon their competency to regulate the emotional climate and to provide necessary support.

### The quest for evaluation

However, in spite of this major investment in the FPS in order to prioritize families with children and violence, no study has been undertaken to explore the aftermath and sustainability of the kick-off project. Limited areas are described, but not the general policy of the partaking offices or the FPS in general (see e.g. Norwegian Directorate for Children, Youth and Family Affairs 2011, 2013). Recently, Middelborg and Samoilow (2014) introduced a treatment perspective on violence in the family in a child focused and child imagined way, with detailed guidelines for conversations with the parents, giving, however, but a few examples of the inclusion of children as partaking subjects. No studies illuminate how the FPSs in more general terms practice the continuation of the knowledge developed through the project.

The unique existence of having a state-financed and sanctioned public treatment agency at a low threshold—the FPS, with a mandate to give priority to specialized treatment for families with children and violence, creates an urge to explore the general ongoing practices after the initiating project. Given such a mandate, how are the services provided when children live in families with violence?

The Tromsø FPS took part in the kick-off project and subsequently aimed to give priority to families with children and violence (Rostadmo 2011). This office is one of the largest FPSs in the country. Therefore, it offered an excellent opportunity to explore this office as an example of ongoing practice in the aftermath of the project. As a newcomer to the FPS in Tromsø in 2010, the first author therefore initiated a study, which was undertaken in agreement with the leadership.

The study asks the following interrelated research questions: What cases are referred to the agency with children living in families with violence and what services are provided? What stands out as main choice points, dilemmas, and challenges in supplying specialized treatment in these cases? How does the FPS practice *violence*-*sensitivity*? And how is the child included and *the psychological child position* taken care of?

The aim of the study is to contribute to the development of a public, low-threshold, specialized family treatment service that best meets the needs of families with children and violence.

## Method

### Participants

Data were all cases at the Tromsø FPS through a period of one calendar year (2012) with children living in families with violence, where violence was reported at referral and/or exposed later. Cases were collected from the total case-load of clinical and Mediation Institute cases, and then cross-checked through the logbook from an internal, weekly quality meeting for all cases with children and violence. 103 out of 554 clinical and 3 out of 336 Mediation Institute cases were included (106). The total number of children was 205, with 58 children below 4 years and 147 from 4 years and above. 33 families had children all below 4 years, 21 families both above and below age 4, and 51 only above age 4. The average number of children inside each family was similar to the rest of the country. The office covered a geographical area of 4.5 % of mainland Norway plus Longyearbyen, with similar ethnicity and the same relative proportion of children below 18 years as the rest of the country.

### Procedure

All professionals (9)—psychologists (2), clinical psychologists (3), social workers and special teachers (4)—of these certified family therapists (5), under family therapy education (1), without such certification (3), completed a semi-structured questionnaire for each of his/her cases for the total of 106 cases. The questionnaire was filled out separately by the professional(s) working in the specific case and anonymized for all except that/those person(s). The questions were developed through the study of relevant literature, consultations with professionals with extended knowledge in the field, and through thorough discussions among all the colleagues at staff meetings about what made up a manageable amount of questions to complete within an acceptable limit of time given the daily pressure of service delivery. Descriptive summary statistics were presented at subsequent staff meetings for the collective explorations of main cross-points, dilemmas, and challenges. The first author was among the clinicians and carried out the work. Areas for exploration across all cases included three extensive topics:

1. What cases are referred?

How many cases have violence reported at intake or later? What type of violence is reported, from whom against whom, and who refers and informs about violence?

2. What services are provided?

What cases get priority with how long waiting time? Who defines that actions are to be called violence and who informs the police and the CPS? What cooperation and conversations are going on? Are steps taken to safeguard clients? Are assessment tools used, e.g. about other problems like psychic health or substance abuse? Is there any connection between the work done and types of violence—for instance the inclusion of children, of other services, number of sessions, or the closure of cases?

3. What stands out as main choices, dilemmas, and challenges and how is the psychological child position taken care of?

The Norwegian Data Protection Authority was consulted, who informed that this study did not require their approval.

### Analysis

Data were analyzed by the Statistical Package for the Social Sciences (SPSS). Descriptive statistics were used to get summary statistics on all cases. Areas for systematization across all cases included the three extensive topics. The Fisher Exact Test was applied to test statistical significance in 2*2-crosstables and the Brown-Forsythe-Test for group differences on the number of conversations. Specifications of main cross-points, dilemmas, and challenges were analyzed and systematized through conversations with all colleagues at the office applying a participatory research approach (Johannessen et al. 2011). This was done by presenting descriptive summary statistics for discussions at three consecutive meetings; at each meeting the discussions from the previous meeting was pursued and expanded in order to get as rich and extensive differentiations as possible of the main cross-points, dilemmas, and challenges. Similarities and divergence in opinions were discussed and summarized conjointly until consensus. In the following the authors first describe what cases are referred, then the provided services, thereafter main choices, dilemmas, and challenges.

## Results

### What Cases are Referred?

#### Information About Violence at Referral or Later

One-fifth of all clinical cases in 1 year are families with children and violence (106 of a total of 554 clinical cases) and 3 Mediation Institute cases (3 out of a total of 336).Violence is reported at referral in 62.3 % of the clinical cases the (66 of 106) and later in 37.7 % (40 of 106). No Mediation Institute case has violence informed at referral.

#### Types of Violence

Physical violence and combined physical and psychic violence are included in more than three quarters of all cases (77.4 % of all cases). Remaining cases are psychic violence. The degree and amount of physical/psychic violence varies from life-threatening actions to knocking, hitting, pressing, pulling over time combined with threats, criticism, and detailed control. Psychic violence is extended use of threats of physical harm, criticism, and detailed control over time.

Looking more closely, there is a statistically significant association between types of violence and whether violence is reported at referral or not (*p* = 0.03). If violence is reported, physical violence is most common. There is also a statistically significant association between physical violence and the request at referral for getting help against physical and psychic violence (*p* = 0.004), but no statistically significant association between psychic violence and types of request at intake.

#### Who Uses Violence and Who are Exposed?

Most frequent offenders are biological fathers, involved in 76.4 % of all cases—acting alone in three-fifths (62.3 %). Mothers alone are offenders in 10 %, but are involved in 25.4 % of all cases. Stepfathers act alone in 5 %. Looking at the total amount where either one or both primary caregivers act violently, this aggregates to ca. 89.6 % of all cases (95 of 106). Children (teenage sons/brothers) offend in 6 of 106 cases.

Most exposed are mothers alone, or mothers and children together (66.1 % of all cases). Children are exposed in all, as main target in 64.1 % of the cases. Fathers are more seldom exposed (15 cases), and if exposed, they are mostly together with others who are exposed simultaneously (13 of 15).

#### Who Initiates Referrals and Who Informs About Violence at Referral?

The family itself is by far the most frequent referrer (78 of 106 cases), both when violence is reported at referral (41 of 66) and later (37 of 40). From inside the families, mothers refer the most. Next comes the CPS, either referring alone (18) or together with the family (8). The CPS refers one-fourth of all cases (26 of 106), with violence usually informed at intake (23 of 26).

Looking more closely at who refers from the family, of exposed and/or offender, the offender takes few initiatives, independent of that person’s role in the family. If the mother offends alone (11 cases), she refers in 2. If together with the father (12 cases), she refers in 3. Also fathers are low referrers, mainly if he himself is subject to the violence (15 of 17 referrals from fathers).

### What Services are Provided?

Here we look at main characteristics of the services provided from intake to discharge.

#### What Referrals get Priority and Bypass the Waiting List?

All cases *with* violence reported at referral, bypass the waiting list (66 of 106). All *without* reported violence at intake go to the waiting list (40 of 106). Referrals from the family dominate both cases bypassing the list (41 of 66) and those going to that list (37 of 40).

#### Time Before First Appointment

A significant difference in waiting times is evident in cases placed on the waiting list versus those that are not. Of cases bypassing the list, 37.0 % gets an appointment within the first week, and almost 68.2 % within 2 weeks. *All* cases with known violence at referral are offered a first appointment within the first consecutive days. *Any prolonged waiting* is due to reasons from outside of the FPS. Typically, cases going to the waiting list have a waiting time of 4 months; the only exceptions are Mediation Institute cases with a mandated delay limit of 3 weeks (3 of 106 cases).

#### Cases Reported to the CPS and/or Police, and by Whom

The majority of the cases are reported to the CPS and/or the police (78 of 106 cases). Only ca. one quarter is not (28 of 106). All cases with no reports to the CPS (36 of 106) are self-referred by the family to the FPS.

Looking more closely at who reports to the CPS, most referrals come from the FPS and the police, thereafter from mothers, only a few from fathers. Others from outside are also important reporters, these are the extended family and private network. In one instance only the *child* contacts the CPS. Cases referred to the CPS most frequently contain physical violence.

Looking more closely at who reports to the police, mothers are the largest category, followed by the CPS. Also here, outsiders are important. *Five children* contact the police directly—alone (2), together with father/mother/school (2), or with the CPS (1). Cases reported to the police contain most frequently physical violence, which is most often reported at referral.

#### Who is the First to Define Violence?

This refers to the one first defining violence independently of whether that case is reported to the police and/or CPS. If reported, these agencies can in their own terms be the first to define that violence is going on. Most frequent definers are mothers (61.3 %), fathers more seldom, and mostly if they themselves are subject to the violence (15 of 17 cases). Also the police and CPS are frequent definers (45.3 %), as well as the extended family/private network—in almost one-fifth of all cases. Additionally, FPS is a main contributor (45.3 % of all cases), most often together with others.

#### Who Initiates Safety Precautions?

Precautions are initiated in 76.4 % of all cases (81 of 106). FPS is the main initiator (55.7 % of all cases). The purpose is to protect the exposed from being more exposed. Precautions are effectuated by the police (23.6 %) and/or the CPS (30.2 %) according to their specific instructions, and/or are elaborated by the FPS in cooperation with the clients and their private network.

#### Who are Cooperating Agencies?

FPS collaborates mostly with the CPS (50.0 % of all cases), the police (8.5 %), and with most relevant public agencies in the field (22.6 % of all cases) such as crisis shelters for women, The Children Advocacy Center, adult psychiatry, hospital/somatic child department, and the judicial system. The least collaboration takes place with the primary health system (3 of 106 cases) and specialty mental health services for children and adolescents (2 of 106 cases). In one fifth of all cases the FPS works alone.

#### What Therapeutic Meetings and Standard Assessments Take Place?

FPS arranges therapeutic meetings which include either adult—single or together as a couple or parents, children separately and/or together with adults, with or without the inclusion of referring services. Here the term “therapeutic meeting” refers to meetings independent of the specific theoretical/methodological approaches applied by the professional. Most meetings are with adults. Children are included in 39 of 106 cases, but in few of the total sessions of these 39 cases (15.2 %). *Almost no child below 4* *years* partakes (4 out of the 39 cases; 4 out of a total of 58 children below 4 years). *No child* is included in 67 out of 106 cases.

Standard assessment tools are used in 22.6 % of all cases to assess experiences and impact of violence and evaluate risk. Problems of substance abuse or mental health are reported as known in 17.0 % of the cases; for the rest, there is reported no knowledge of such issues. The tools consist of an extensive cluster of internationally elaborated measures for trauma, abuse, and violence exposal, sequels, and risk—a cluster collected and made available by the mentor institutions of the kick-off project (Kartleggingspakke ATV-SfK 2008).

#### Number of Sessions and the Closing of Cases

Mainly, services are brief: most common are 7 or fewer sessions (70 % of all cases), 84 % of the cases get at most 12 sessions, the remaining cases get up to 49 sessions. Looking more closely, there is a statistically significant higher number if *both mother and children are exposed* to violence compared to mother alone or not mother (*p* = 0.02), and *if children are included* *into the work* compared to when they are not (*p* = 0.01).

The closing of cases (76 closed and 30 not closed) suggests that cases last longer when both child and mother are exposed and combined violence happens than if mother alone is exposed to one type of violence. But these differences are not large enough to be statistically significant.

#### Differences Across Professionals

A distinct difference appears among professionals concerning the inclusion of children: *those with the prior most extensive therapeutic practice with children* include children far more often, both concerning the total number of cases and the total number of sessions in each case; and if children participate, the number of sessions grows, and, subsequently each case consumes more time. This difference is independent of the professionals being certified family therapists or not.

### Main choices, Dilemmas, and Challenges

In the following we note and discuss main choices, dilemmas, and challenges as analyzed through the participatory research approach. Eight areas are outlined. We focus on what this can tell about providing *both a child focused and a violence*-*sensitive family treatment service.* The elaborated recommendations are highlighted by italics.

#### A Public FPS Can Succeed in Giving Fast Priority When Violence is Reported at Intake

Most of all, this study tells that *if* a Norwegian FPS, as a public, specialized treatment service, gives priority to families with children and violence, *a great amount* of *the total case*-*load* becomes exactly so—here one fifth of all clinical cases in 1 year. Every fifth case is a large number, considering the open and free of charge access for all types of family- and relational problems. Moreover, the study shows that the same FPS *can manage to live up to* a political mandate of supplying both priority and short waiting time when violence is reported at intake. *All* cases with known violence at referral are offered *a first appointment* *within the first consecutive days*. *Any prolonged waiting* *is due to reasons from outside of the FPS*. *Succeeding* with such a goal is surprising, since the office—like most FPSs in Norway, serves a large geographical and population area with a major pressure of other cases.

Such success can be obtained only through professional dedication and a clear leadership. And it depends on political priority. Because the practice has *a major drawback*. The priority creates a queue. Other relational problems—like couple therapy and complicated family relations—have to wait, which is in conflict with the aim of prevention by early service that counts as a target for the same service (Norwegian Directorate for Children, Youth and Family Affairs 2014).

On the other hand, the proportion of cases *with no reports of violence at referral is large*, 38.6 %, going to the waiting list with long delay. This later emergence of violence *may* indicate a service providing violence-sensitive collaboration. It may, however, also point to shortcomings in the intake routines, the practice being too imprecise to invite issues of domestic violence. As stated by Posada and Pratt (2008) and as outlined by Todahl and Walters (2011) on the basis of a systematic review of screening practices of partner violence, family therapists as helping agencies have a unique professional possibility to examine the role of domestic violence in their work. Accordingly, they recommend acknowledging the great and unique social and professional responsibility of these agencies to see and hear domestic violence. In line with these suggestions, the large amount of late reporters of violence seen in this study underscores *the benefit of including violence*-*specific questions as part of an ordinary intake procedure in family treatment services*.

#### The Home as the Central Arena of Safety and Growth is Affected in all Cases

Second, in line with a national survey of the prevalence of violence in Norway (Thoresen and Hjemdal 2014), mothers or mothers and children are the most exposed to all kinds of violence. Fathers are almost exclusively exposed to violence when together with others who also are directly exposed. Children are affected in all cases, some of them as offenders. On the other hand, abusers are mostly fathers (76.4 % of all cases), or stepfathers (5.8 %), but *also* mothers offend—alone or involved with others (25.5 % of all cases). In total, one or both of the primary caretakers are offenders in 89.6 % of all cases. Thus, consistent with Øverlien (2012), *the home as the central arena for safety and growth is affected by domestic violence in all cases*.

Such knowledge suggests that family therapists should expand on a more traditional view of domestic violence characterized by male perpetrators. Instead, in line with Stith et al. (2012) and George and Stith (2014), it seems necessary to be open to the fact that, although men and fathers are by far the most dominant abusers, both mothers and children use violence. As also stated by Allen (2012), recent research makes it necessary *to be open to include* other participants’ contributions in domestic violence.

#### Families and Mothers Refer the Most

Third, the study shows that the most dominant referrer is *the family itself*, both when violence is reported at intake (62.1 % from the family) and underway (92.5 % from the family). From inside families, *mothers* are most frequent referrers. In total, mothers refer the most both to CPS, police, and FPS, while fathers refer much less, and almost exclusively when he himself is subject to the violence. Of all referrals, physical violence is most frequently reported both in the referrals to the FPS and later as reports from the FPS to the CPS and police.

Compared with the fact that many *never* tell about experiences of violence despite major sequels (Thoresen and Hjemdal 2014; Tracy and Johnson 2006), that violence is often minimized by the exposed because events are too painful to process or too shameful to tell (Siegel 2013; Tracy and Johnson 2006), combined with the fact that offenders themselves often play down and minimize violence (Adams 2012), this high amount of family referrals sends a main message: giving priority from a FPS to families with children and violence provides a public service that the *families utilize*. It creates a place for people to dare to address questions of possible doubt, shame, and silence, *without* necessarily having to inform about violence as a required entry ticket, or having to wait for obvious signs of trauma. *They can come, taste, evaluate*—*and dare*.

However, substantial studies underscore that *more knowledge is needed in the general society about consequences for children* in order for both offenders and caregivers to ask more easily for help (Adams 2012; Askeland et al. 2012). In accordance with Raundalen (2007) and Wekerle (2013), an extended perspective is required on “childhood as having its own value and its own rights”, which means to realize that to ask for assistance to change violence is not exclusively for the benefit of the adults, but as an imperative and a need for the child itself. As the study tells, mothers refer; fathers need more hope and faith to see and dare. And, as we will see below, offenders of both sexes need more understanding of the consequences of domestic violence for their children, to nourish necessary willingness and courage to change.

#### Offenders Need Others as Promoters for Change. Children Depend on Adult Advocates

Four, across all cases, *the one who acts violently refer the least, no matter who that person is.* If the mother acts alone (11 cases), she refers in 2. If together with the father (12 cases), she refers in 3. Also fathers are low referrers if offending (17), and then mainly if he himself is subject to the violence (15 of 17). Thus, the driving force for change is the ones exposed. The one wearing the shoes, who knows where it hurts, is the one to call for change. *Except for the child*: only *one* child contacts the CPS and only *two* the police. When children otherwise initiate (3 out of 6), they call persons from outside the close family.

Again is illustrated, children are dependent on grown-up advocates and spokespersons. The ones executing violence need others as *prime motor* for change. The clear-sightedness and understanding of a necessity for change is unevenly distributed when violence happens. Recently, research from using client feedback to improve therapy (Duncan and Sparks 2008), also in a FPS naturalistic setting (Anker et al. 2009; Sundet 2014; Ulvestad et al. 2007), shows the importance of clients’ feedback for the therapeutic processes to be useful for necessary changes. This study underscores *the importance of inviting the most silent voice*—*the child*—*into the treatment process, to inform and form that process to safeguard needed changes.*

#### FPS Succeeds in Providing an Open Route for Collaboration with the CPS and Main Public Services

Five, a FPS, by its priority, can succeed in providing an *easy accessible and much used route for the CPS to refer families with children and violence to specialized treatment*. The study shows that the CPS is the most extensive referrer to this FPS, next to the family, delivering one fourth of all referrals, and usually informing about violence at intake. Each case thus informed, gets immediate appointment. Moreover, the other way around, the FPS reports approximately one fifth of all cases to the CPS. In sum, this makes up *a fluent two*-*sided collaboration between these two important public services, the CPS and the FPS*. Additionally, a FPS, by its priority, *also brings about an extensive collaboration with other relevant agencies, including the police*. Least cooperation takes place with the primary health service with but a few links, and the specialty mental health service for children and adolescents, with almost no cooperation.

Considering the studies documenting the overall difficulties in the Norwegian specialty mental health service to perceive children’s experiences of violence, combined with this service’s reluctance to include children’s family in ongoing work (Ormhaug et al. 2012; Reigstad et al. 2006; Reigstad 2012; Røberg 2011; Wassnes 2012), as well as refusing referrals for children exposed to violence because they did not have a diagnosis and/or had too unstable caring situations (Heltne and Steinsvåg 2010, 2011), the present study again underscores the challenge of providing such services to families with children and violence. Given *both* the great amount of families referring themselves to the FPS when violence happens, *and* the many cases where violence is disclosed after referral, *along* with the research documenting the sequels for children of domestic violence, *an easier access is called for, to the specialty mental health service as well as a more fluent collaboration with the FPS*.

Moreover, the low frequency of collaboration with the primary health service sends an additional message: In line with recent voices from the Norwegian primary health field, urging to include questions about family violence into standard assessment procedures (Ude 2014), the present study amplifies the need for *an earlier recognition of violence in the primary health service as well as a more extensive inclusion of the FPS* *as part of their service delivery.*

#### FPS Provides Brief Specialized Treatment

Six, this public FPS, by its priority, manages to deliver *brief treatment services*. Approximately 70 % of all cases get 7 or fewer sessions, and 84 % get at most 12 sessions. More sessions (from 13 to 49 sessions) happen mainly *when children are included and when both mother and children are exposed*. Thus, considering the research on consequences of violence on mental and somatic health, the study suggests that *specialized treatment* *services can be brief if delivered at the right time*—*at an easy accessible place, with a low threshold, when the need for help is wanted and experienced as urgent*. Economic costs can diminish both for society and single persons, since violence has a high cost—in Norway between NOK 4.5–6 billion per year (Rasmussen et al. 2012).

Consistent with studies on cost-effectiveness of the practice of marriage and family therapy (see e.g. Crane and Christenson 2012; Crane and Payne 2011; Gelles and Maynard 1987; Klientz et al. 2010; Moore et al. 2011), the present study shows a relatively inexpensive modality of psychotherapy. However, far more thorough outcome measures are necessary. Although Partners for Change Outcome Management System is underway in the FPSs (Anker et al. 2009; Duncan and Sparks 2008; Sundet 2014), outcome measures in this study were not systematically completed. *A systematic use of such measures is required for accounts of effect*.

#### The Work is Violence Informed, But Includes Spare Use of Standard Assessment Tools

Seven, only in 22.6 % of the cases are standard assessment tools used to assess experiences and impact of violence and to evaluate risk. Supplementary violence-informed focus takes place by the CPS referrals containing detailed reports of violence (25 % of the cases) and by FPS reports to the CPS (20 %), which lay the ground for extensive violence-informed cooperation between CPS and FPS, in addition to reports to and collaboration with the police.

However, such low-frequency use of assessment tools stands out as a challenge for several reasons: Substantial documentation shows that violence is frequently under-communicated (Askeland et al. 2012), minimized both by the exposed (Siegel 2013; Tracy and Johnson 2006) and by the offender (Adams 2012), and also linked to strong feelings of parental shame when children are included (Holt 2014). A low-frequency use of standard assessments tools is especially challenging considering the family therapists’ unique possibility to be the ones to examine the role of family violence as part of therapeutic collaborations (Posada and Pratt 2008; Todahl and Walters 2011).

Overall, there has been little published research to document how, or if, assessment tools are utilized by marital and family therapists (Stith et al. 2012). However, many studies have offered attempts to strengthen an integration of family assessment and intervention models (e.g., Asen et al. 1989; Bentovim 2004; Fernandez 2007; Cohen and Mannarino 2008; MacGregor et al. 2014; MacMillian et al. 2013; Schacht et al. 2009; de Melo and Alarcáo 2011). In general, although assessment tools need elaboration in contexts of violence-sensitive collaborations, a low frequency use may diminish a necessary respect for the need to be informed by all involved in suitable and safe enough contexts.

A similar dilemma appears for substance abuse and mental illness questions. Problems of substance abuse or mental health are reported as known in 17.0 % of the cases; for the rest, there is reported no knowledge of such issues. Recently, a growing understanding has emerged of violence often co-occurring with other significant problems, particularly substance abuse. A large body of research has found a relationship between domestic violence and substance abuse in both clinical and nonclinical samples (Christensen 2010; Donohue et al. 2006; Stith et al. 2012). Consequently, this study indicates an under-consumption of *standard assessment tools necessary to provide a sufficient violence*-*sensitive FPS*.

#### Too Few Children are Invited

Eight, surprisingly considering the specific mandate to focus on families with children and violence, services are *mainly offered to adults*. Only 39 out of 106 cases include children, and only a few of the total sessions of these cases (15.2 %). *Almost no* child below 4 years takes part (4 out of 39 cases; 4 out of a total of 58 children below 4 years)—although more than half of the cases (54 out of 106) have children below 4 years. Given the consequences for children of domestic violence—including for children below 4 years (ACE-study 2013)—this stands out as *an alarmingly low rate*. The study shows that also a service with a specific priority for families with children and violence includes the child far too rarely.

Thus, the practice illuminated in this study coincides with voices from the clinical research and literature pointing to family therapy providers having been too hesitant to include children (Hartzell et al. 2009; Lund et al. 2002; Rober 2008; Ruble 1999; Sori and Sprenkle 2004). However, the study shows a distinct *professional difference*: independent of professionals being certified family therapist or not, the ones most experienced in therapeutic work with children, include children far more often; and *if children participate, the number of sessions grows and subsequently consumes more time*. Accordingly, a new question comes up: Given the great impact of violence on children, how can the service bring about *a more de facto inclusion of the child*?

## Discussion

### Priority and Collaboration

Considering the unique existence of having a state-financed and sanctioned public treatment agency at a low threshold, the FPS, with a mandate to prioritize specialized treatment to families with children and violence, this study conducted in one of the larger FPS in Norway, shows first and above all that *if* such services get priority, these cases are *flooding in*. Most of all, it opens for *people themselves to come and ask for assistance*. *It opens doors for people living in the midst of violence.* Moreover, the study elucidates that *it is possible* for a public FPS to fulfill a mandate to provide fast-track services when violence is known at intake, and to supply *a direct route for the CPS* to get coordinated, specialized treatment, as well as collaboration with other main public agencies.

In sum, the study indicates that the investments made through the national project “Children Living with Violence in the Family” *shows a promising start*. It shows that it is possible for a public FPS to *provide a direct, much used and efficient route both for private persons and cooperating agencies for collaboration and specialized treatment.* It exemplifies a *possible way to fast*-*track family therapy services when violence happens*.

### Both Family Therapy and Taking a Stand Against Violence

Moreover, the study illustrates a FPS that is not afraid to take part in understanding and defining actions as violence, and to initiate necessary safety precautions. In short, it shows a public FPS that manages *to take a standpoint against violence*. Such a FPS becomes an active collaborator with both private persons and main public agencies—mostly the CPS and police. Thus, the same FPS exemplifies a road that openly combines therapy with taking a stand against violence.

Such a combination bypasses the strong and general warnings from feminist-informed viewpoints that the family therapy field is minimizing power differences between men and women inherent in family violence. The field has been accused of providing an either-or approach, where violence is concealed for the profit of reconciliation (Stith et al. 2012) in combination with a too low-frequent use of assessment tools to recognize violence (Schacht et al. 2009). This FPS’s extensive collaboration in (1), defining violence, and (2), initiating safety precautions, exemplifies a *“both*-*and” approach*.

Simultaneously, a main challenge remains to strengthen the use of standard assessment tools. The request to realize the unique responsibility of family therapy services to thoroughly examine the role of domestic violence as part of treatment (Posada and Pratt 2008; Todahl and Walters 2011), is most relevant for this FPS.

### The Most Silent Person as the Ultimate Litmus Test

However, an overruling phenomenon is apparent: almost no offender asks for help. The one exposed to violence, is the one asking for assistance—except for the child. All children are affected; almost none contacts any helping agencies. Because the offender needs others as promoters for change, to include experiences of the exposed into ongoing work, becomes crucial. In line with Per Isdal’s (2013) definition of violence, the one met by violence is *the most important measure of change*—that good enough work is done and necessary changes worked out. Subsequently, *to include that person*’*s account in ongoing work brings about the utmost litmus test for ensuring that sufficient work is done for families with children and violence*. However, as the study illuminates, this turns out to be the dominant shortcoming of the services provided.

### The Absence of the Child

Because, paradoxically, the study elucidates that even a FPS with a precise priority to include a child perspective into family violence work, *runs with a dominant lopsidedness*: Violence *is* absolutely an issue. But although children are affected in all cases, treatment services are offered *almost exclusively* to the adults. Out of a total of 106 cases, only 39 families include children and only in 15 % of the total sessions in these families. Only 4 of them include children below 4 years. Given the substantial clinical research and documentations of the consequences of domestic violence for children, this sums up as *a major neglect of the child*. *The living, partaking child is to a large degree excluded and the psychological child position not adequately taken care of.* The absence of the child’s specific experiences conceals necessary insight into the impact of violence, and reduces the possibilities of dialogically informed changes for those involved. In line with Raundalen’s warning at the end of the kick-off project, this study from a large FPS shows a still ongoing and general risk when working with families and violence, that the service becomes “softhearted on behalf of adults, and hard-hearted on behalf of children” (Norwegian Directorate for Children, Youth and Family Affairs 2011).

### The Rights of the Child

In a Norwegian judicial context, children’s rights are strengthened by the UN Convention on the Rights of the Child being incorporated into Norwegian law by an amendment in 2003 to the Human Rights Act, which is given precedence over any other legislative provisions that conflicts (Act relating to the strengthening of the status of human rights in Norwegian law (The Human Rights Act) 21.5 (1999)No. 30.). This human rights approach to child protection constitutes the central catalyst for a paradigm shift to transform both child protection and participation (Wekerle 2013). A child’s rights paradigm is “the declaration of the child as a right holder and not as a beneficiary of benevolent activities of adults” (Article 13, Para. 72b); it constitutes premises for the inclusion of children.

In more details, according to the Convention’s Article 13: “States Parties shall take all appropriate legislative, administrative, social and educational measures to protect the child from all forms of physical or mental violence, injury or abuse…” Article 12 says: “States Parties shall assure to the child who is capable of forming his or her own views the right to express those views freely in all matters affecting the child, the views of the child being given due weight in accordance with the age and maturity of the child.”

However, even for a country with the best of intentions, with a ratified UN Convention, with significant departmental plans and major measures taken to safeguard the child, the present study tells that the experiences and costs of the child when family violence happens are still almost not included into the work of this important public, state-organized family treatment service—despite its specific mandate to do so. *Children still “fall through the cracks”.* *A* *major neglect of the child is taking place.* The political mandate calls for *a sharper look* at how the weakest part—the child—is taken care of and not been thrown “out with the bathwater”.

### A Triple Viewpoint: Rights of the Child, Violence, and Family Therapy

All the more surprising is an absence of the child, knowing that children themselves, if given opportunities, want and consider it crucial to be invited into sharing and understanding when violence happens (Ernst 2006; Flåm 2013; Flåm and Haugstvedt 2013; Jensen et al. 2005; Ungar 2004; Øverlien et al. 2009). And they overwhelmingly want to be involved in family therapy sessions when asked (Hartzell et al. 2009; Sheinberg and True 2008; Stith et al. 1996; Fauske 2011). Moreover, children find it frustrating if they are kept from participation either by being left in the waiting room or by being asked to participate in an adult-oriented process that do not include appropriate avenues for their participation (Stith et al. 1996).

However, as outlined by Vis et al. (2011), to engage children in collaborations and decisions affecting their lives, and for that participation to be helpful, sets standards for ongoing work: it calls for inviting children into contexts that provide information, explaining what is happening, and to be open to children’s own agendas and questions. Because although invited, children do not necessarily join: children investigate, move, and remove from attending according to their own experiences of being properly attended to (O’Reilly and Parker 2013). If asked, children give advices to what makes them feel included: to be accepted and allowed to express their own feelings and that therapists adjust to each person and give space for various perspectives (Hartzell et al. 2009). In short, children themselves are active researchers of ongoing dialogical avenues and possibilities (Bråten 2007; Flåm and Haugstvedt 2013).

As stated by family therapists themselves, to involve children may bring them to the limits of comfort, leading away from well-known approaches with adults into avenues of perhaps more unknown ways of talking, telling, and sharing (Hartzell et al. 2009; Lund et al. 2002; Rober 2008; Wilson 2008). However, if done, also caregivers regain better recovery after family violence if therapy is provided for their children (Holt et al. 2014) as well as for children and parents combined (Chaffin et al., 2004; Herschell et al. 2000; Herschell and McNeil 2005).

Although the focus of this study is not an elaboration of how to involve children into family and network-oriented work, recent clinical literature and research provide ample suggestions (see e.g. Chaffin et al. 2011; Cohen and Mannarino 2008; Grammer 2009; Herschell and McNeil 2005; Kolko and Swenson 2002; Kjellberg et al. 2013; Larner 2003; Lowenstein 2010; Lund et al. 2002; MacMillian et al. 2009; Rober 2008; Sheinberg and True 2008; Siegel 2013; Sori 2006; Swenson et al. 2010; Turns and Kimmes 2014; Vetere and Dowling 2008; Wilson 2007, 2008). As stated by pioneers in the field, a better understanding of attachment processes between parents and their children help guide a better treatment for maltreated youngster (Cicchetti et al. 1989). But warned by other forerunners, attachment lenses may contribute to mask the child’s needs for differentiated support, disguising an overall responsibility which goes beyond the goals of reducing maltreatment by parents as a “partial solution”, and calls upon a closer look at the needs of the child (Graciano and Mills 1992).

Across approaches, as the present study underlines, a triple viewpoint is needed: to include topics of violence, to include the child, and to explore room for dialogues among children and adults.

### Integrative Family Perspectives are Called For

In sum, the fact that in all cases of this study the home as the central arena for safety and growth is affected, gives guidelines for future practices: since *violence creates asymmetry*, perspectives are called for that includes perspectives on *both* the child, *and* the adult exposed, *and* the abuser. To maintain a limited single person perspective, or solely a couple or parent perspective, or a more floating family perspective becomes restrictive.

Approaches are called for that promote and integrate *both* the uptake and use of intimate partner violence *and* child maltreatment knowledge (MacGregor et al. 2014). That means to include and integrate involved voices not solely conceptually but in vivo and de facto to inform needed changes.

As outlined by Stith et al. (2012), who offer a detailed review of the current state of the relationship violence literature, a major turn is needed in the domestic violence therapy field away from more individualized treatment perspectives towards family oriented approaches. And as stated by Siegel (2013), from a detailed review of the research in the field of family violence, services offered to families with violence have not kept pace with the emerging research providing extensive information about the sequels of family violence; most frequently, treatment has been offered as separate services to either the one or the other adult part, and too rarely in conjoint treatment, even though the rationale and indications for efficacy have been repeatedly stated for an expanded approach to treatments that incorporate family systems and the persons involved.

Looking into the future, the Norwegian FPSs will continue being a gateway for cases asking for treatment for crisis and relational problems. Many families with children and violence will enter into the FPS, where working with violence will require thorough intake practice, violence-sensitive follow-up and fluent cooperation. Fortunately, this public service *already has* a politically mandated priority for cases with children living in families with violence (Norwegian Directorate for Children, Youth and Family Affairs 2014). Therefore, a FPS more *prepared* for including children is needed. In line with the ACE-study (2013), an opening for children’s voices when violence happens provides the strongest means to eliminate the misuse of power and the loneliness hidden in secrets and silence—and to open doors for change. Thus, a FPS with priority for children and families with violence constitutes a key to *better general public health both in the short*- *and long*-*terms*. Adults need to find such a service. Children have individual rights to get it (Lassen 2013).

Consistent with MacMillian et al. (2013) who take a close look at children’s safety in domestic violence cases, and with Schacht et al. (2009), examining couple therapists’ assessment practices, the present study tells that *integrative family perspectives are called for, which combine violence*-*sensitivity with safety precautions, including the child*’s *partaking voice and position.*

### Limitations

It can be argued that since the data used comprised the professionals’ own evaluation of main choices, dilemmas and challenges, the information presented might be misleading. First, the professionals may be influenced by their own methodological preferences and therefore might not give representative answers. However, since the answers were analyzed conjointly on the basis of descriptive summarized statistics, skewed presentation can be more easily corrected than from single presentations. Second, it may be argued that the lack of information about the concrete therapeutic practices of each professional may blur necessary insight into how divergent therapeutic approaches may influence. Certainly, the study could have been expanded by supplying a more detailed knowledge of each professional’s concrete therapeutic practice, as a supplement to the actual one. Given the nature of violence, studies of detailed therapeutic practices are highly relevant, and could add valuable knowledge to guide future practices in the field. However, such detailed focus did go beyond the time and economic limits of the project. Third, the lack of pre-post measures as evidence of effect can be considered a major drawback, disguising a possibility of unsuccessful therapeutic work. Subsequently, measures in more details to assess risk factors and risk circumstances, and possible changes of these, would give valuable in-depth knowledge of changes. Certainly, a stronger future inclusion and completion of outcome measures in the FPS will provide needed evaluation knowledge. Finally, a more detailed study of the cooperation between the FPS and its closest cooperating agencies, e.g. the CPS, could have been expected, as well a more thorough description and discussion of useful therapeutic approaches based on research and clinical literature for the inclusion of children into family therapy. This is, however, not the aim of the present study.

## Conclusion

The overall message of this study is that the investments made through the national project “Children Living with Violence in the Family” in the FPSs in Norway *shows a promising start*. It illuminates that it is possible for a public FPS to *provide a direct, much used and efficient route both for private persons and cooperating services for specialized treatment and collaboration.* It exemplifies a *possible way to fast*-*track family therapy services when violence happens*. Thus, the study shows that the unique existence of having a state-financed and sanctioned public and specialized treatment agency at a low threshold—the FPS, with a mandate to prioritize treatment for families with children and violence, *has laid the ground for a practice according to intended goals*.

However, while services are provided fairly quickly when violence is reported, the service given calls for changes in several ways: A more violence-sensitive intake procedure is called for, a more fluent and stronger cooperation with both specialty mental health service and primary health service is needed, the use of standard assessment tools is too low-frequent, and outcome measures need a major strengthening to document whether treatment is successful and if violence has been eliminated. Given the nature of violence, particularly follow up measures are required. However, first and foremost, the study calls for a better inclusion of the child. In family therapy, this means talking not solely *about or on behalf of* the child. It means *talking* *with.* It asks for *“with*-*ness” work, more than about*-*ness work* (Anderson 1997; Shotter 2010, 2012). It asks to enlarge the space and means for sharing, and telling *in ways other than those most common with adults,* suited to children’s own age, and capability—to let them share, dare, and thus inform needed changes—without masking adults’ responsibility.

To see the child is inherent in the Norwegian political mandate for the FPS to prioritize families with children and violence. Provoking, however, according to the UN Convention Article 13, to focus the child is required not solely “as a beneficiary of benevolent activities of adults”, or as an ethic of hospitality (Larner 2003). Most important, the main obligation is for the child “as a rights holder in its own right”. Thus, according to the same Convention, the Norwegian FPSs—as a family treatment service of a country complying with this Convention, is obliged to strengthen its efforts to take the child more successfully into account when domestic violence happens. A more de facto inclusion of the child is needed to provide *family*-protection according to the mandate, and not *adult*-protection with only *a side*-*glance at the child*. Subsequently, this study shows reason for and may give push-off to *a prolonged child focused investment* to build the necessary knowledge, therapeutic means, professional courage, and evaluation in the FPSs for a better de facto inclusion of children in cases with family violence.
